# Hemodynamic considerations for prenatal treatment of severe Ebstein anomaly with circular shunt based on two cases and review of the literature

**DOI:** 10.1007/s00404-025-07990-z

**Published:** 2025-03-12

**Authors:** Adeline Walter, Ruben Plöger, Florian Recker, Boulos Asfour, Nathalie Mini, Annegret Geipel, Ulrich Gembruch, Brigitte Strizek

**Affiliations:** 1 Department of Obstetrics and Prenatal Medicine, University Hospital Bonn, Venusberg-Campus 1, 53127 Bonn, Germany; 2 Department of Pediatric Cardiac Surgery, University Hospital Bonn, Bonn, Germany; 3 Department of Cardiology, Paediatric Heart Centre, University Hospital Bonn, Bonn, Germany

**Keywords:** Ebstein anomaly, Circular shunt, Intrauterine therapy, NSAID, Fetal echocardiography

## Abstract

Prenatal severe Ebstein anomaly might be complicated by a circular shunt. In these cases, persistently elevated right atrial and venous pressure (due to severe tricuspid regurgitation) is complicated by a systemic ineffective blood shunt via a DA, resulting in diminished end-organ perfusion and acidosis, due to overall low cardiac output. Affected fetuses are at a significantly higher risk of intrauterine fetal demise. Reduction of ductal flow by prenatal treatment with nonsteroidal anti-inflammatory drugs has recently been described as a potential treatment option. However, published data are limited and management during the antenatal course is not well defined. We provide a literature review to propose a possible algorithm for prenatal assessment and initiation of treatment.

## Introduction

Most fetuses with Ebstein anomaly show only mild or moderate displacement and regurgitation of the tricuspid valve, which does not significantly alter fetal hemodynamics.

**Fig. 1:**
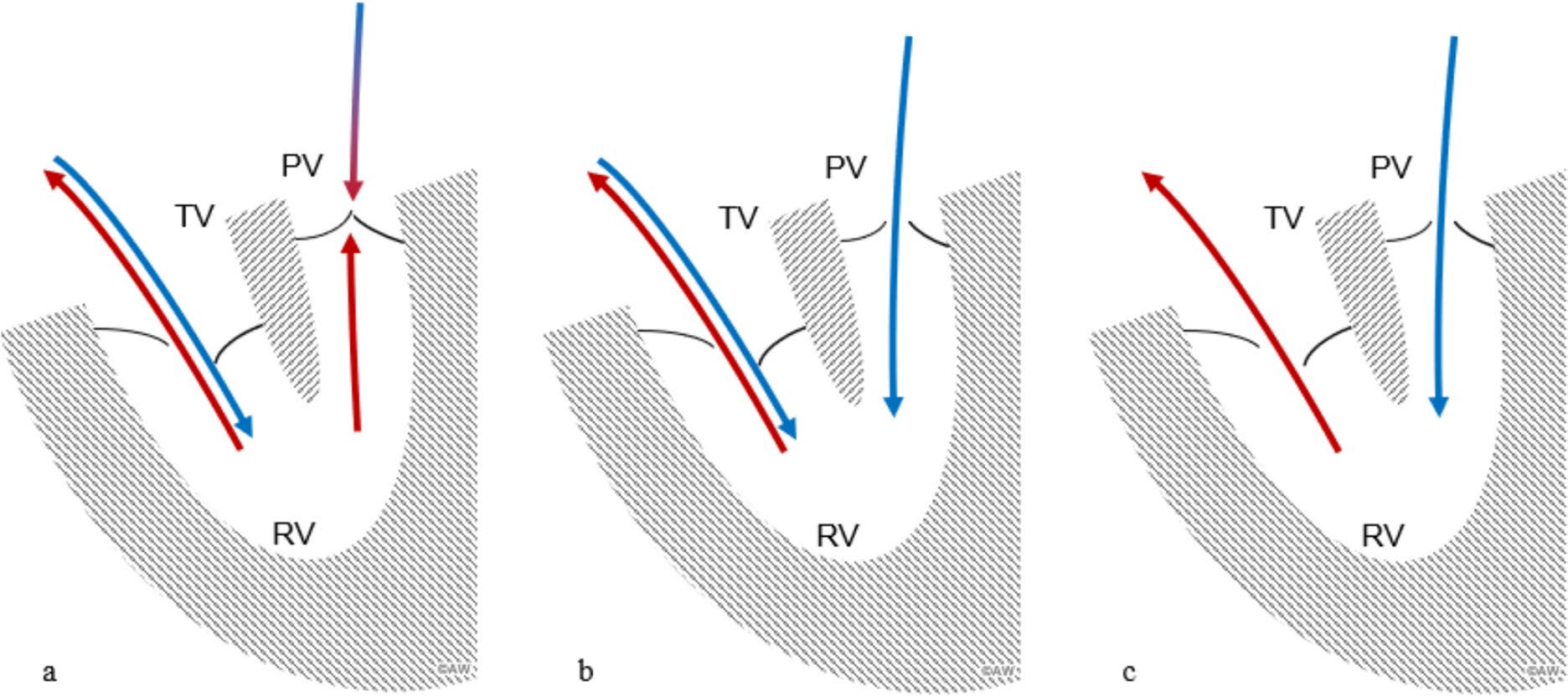
Three different subtypes of a circular shunt are shown. In **a** A functionally pulmonary atresia is demonstrated. Diastolic right ventricular filling in these cases is completely maintained via the *RA* (right atrium). In systole generated ventricular pressure is of systemic level pressure and equal to the high pulmonary vascular resistance in the lungs. Leaflets of the pulmonary artery therefore do not open (black bar). Blue arrows represent the diastolic blood flow, while red arrows represent systolic blood flow. In **b** (incomplete circular shunt), RV is filled via the diastolic *PI* (pulmonary insufficiency) combined with a diastolic filling thru the RA via the tricuspid valve. In **c**, a complete circular shunt is demonstrated, with the RV being entirely filled via the diastolic PI (pulmonary insufficiency), leading to a significant amount of hemodynamic relevant shunted blood. *TV* tricuspid valve, *PV* pulmonary valve

Severe Ebstein anomaly, however, can lead to a life-threatening condition for the fetus, known as a circular shunt [1–3]. In this scenario, blood flows from the right ventricle back into the right atrium, due to significant tricuspid valve regurgitation (TR) [4]. As usual, blood passes right to left across the foramen ovale (FO), flows through the left heart chamber, and into the ascending aorta. In the aorta, however, blood flow is redirected through the ductus arteriosus (DA) toward the pulmonary valve [4, 5]. If the right ventricular (RV) pressure fails to match that of the pulmonary trunk, functional pulmonary atresia (PA) develops. In these cases, the pulmonary leaflets do not open, and the RV is exclusively filled via the RA. This incomplete circular shunt leads to an insignificant systemic steal and hemodynamic consequences are generally well tolerated by the fetus (Fig. 1).

However, as pregnancy progresses and the volume and pressure load of the ventricles increase, the RV may generate even less pressure than in the pulmonary trunk, leading to a pulmonary regurgitation (PR) [4, 6–9]. A complete circular shunt is present, when blood flows left to right through the DA, bypasses the pulmonary capillaries, and re-enters the RV, creating the vicious cycle [3].

The extent of right ventricular filling via the PR competing with the diastolic right atrial filling characterizes the different subtypes of a circular shunt (Fig. 1), with its hemodynamic relevance being mainly explained by the following two aspects [3, 6, 8]: First, progressive volume overload of the RV increases end-diastolic filling and central venous pressure, leading to increased extravascular fluid shunting and severe reduction in lymphatic drainage from the interstitial compartment; in some cases, further increase in venous pressure occurs due to decreased ventricular interdependence caused by massive dilatation of RV and RA. [10]. Second, systemic steal by ineffective blood flow via the circular shunt results in functional low-cardiac output failure with impaired perfusion of placenta and end organs, metabolic acidosis, or intrauterine demise [7].

To interrupt this cycle and prolong pregnancy, maternal NSAID administration has been used to constrict the DA. However, the lack of standardized protocols and safety data limits its application [3, 11, 12].

We conducted a systematic literature review and presented two new cases. Our goal was to outline prenatal characteristics and hemodynamic changes, aiming to develop a potential algorithm for prenatal assessment and treatment initiation, taking into account ventricular function and hemodynamics of the circular shunt.

## Cases

**Fig. 2:**
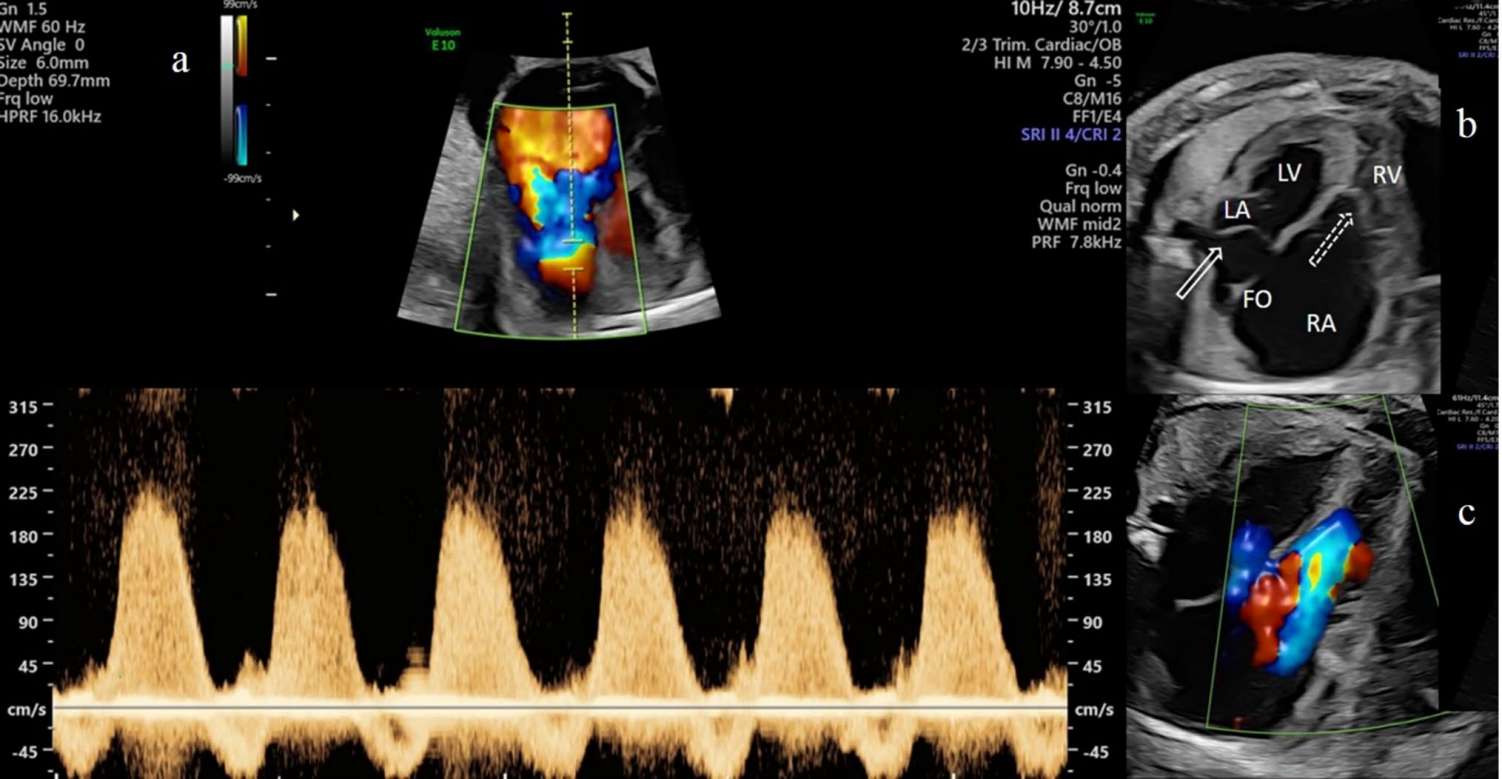
Case 1: **a** Holosystolic tricuspid regurgitation with peak velocity of nearly 230 cm/s shown in a fetus with severe Ebstein anomaly at 31 + 6 weeks of gestation. Color contours appears as a “hot air balloon”. **b** Apical four chamber view in grey scale demonstrates the severe cardiomegaly with the typical apical displacement of the tricuspid valve (open dotted arrow), with a FO (foramen ovale) flap nearly filling the entire LA (left atrium) (open arrows). The RA (right atrium) is dilated including functionally atrialized part of the right ventricle (Celermajer index: 3.23). **c** Color Doppler shows the anatomic origin of the regurgitant jet arising deep in the anatomical RV

Case 1: At 31 + 6 weeks of gestation, hydrops fetalis (pericardial effusion, ascites, placentomegaly, and polyhydramnios) caused by a severe Ebstein anomaly and circular shunt was first diagnosed at our department. There was extreme cardiomegaly, presenting as a “wall-to-wall” heart with a cardiothoracic area ratio (CTAR) of 0.84 (Fig. 2).

Apical displacement of the septal and posterior leaflets of the tricuspid valve resulted in a small functional right ventricle (RV) (Fig. 2). The atrialized inlet portion of the RV and right atrium (RA) showed severe dilatation (Celermajer index 1.85) [13], resulting in ventricular septal paradoxical movements. Doppler echocardiography showed a holosystolic tricuspid valve regurgitation with a peak velocity of 230 cm/s corresponding to a transtricuspid pressure gradient of 22.49 mmHg and a change in pressure over time (dP/dt) of 212.4 mmHg/s (Fig. 2). Further, reverse flow in the DA resulted in exclusive filling of the right ventricle by a severe PR with a peak velocity of 250 cm/s seen in systole and diastole. Umbilical artery (UA) Doppler flow showed a pulsatility index (PI 1.1; 85th centile) with positive end-diastolic flow. The venous duct showed raised pulsatility (PIV 0.87; > 99th centile); in addition, the systolic peak velocity was slightly lower than the diastolic peak velocity (S/D ratio: 0.90) with a deep v-wave, both typical findings in fetuses with severe tricuspid insufficiency [14]. Based on clinical features, the diagnosis of a circular shunt with a high proportion of ineffective blood flow was made.

**Fig. 3:**
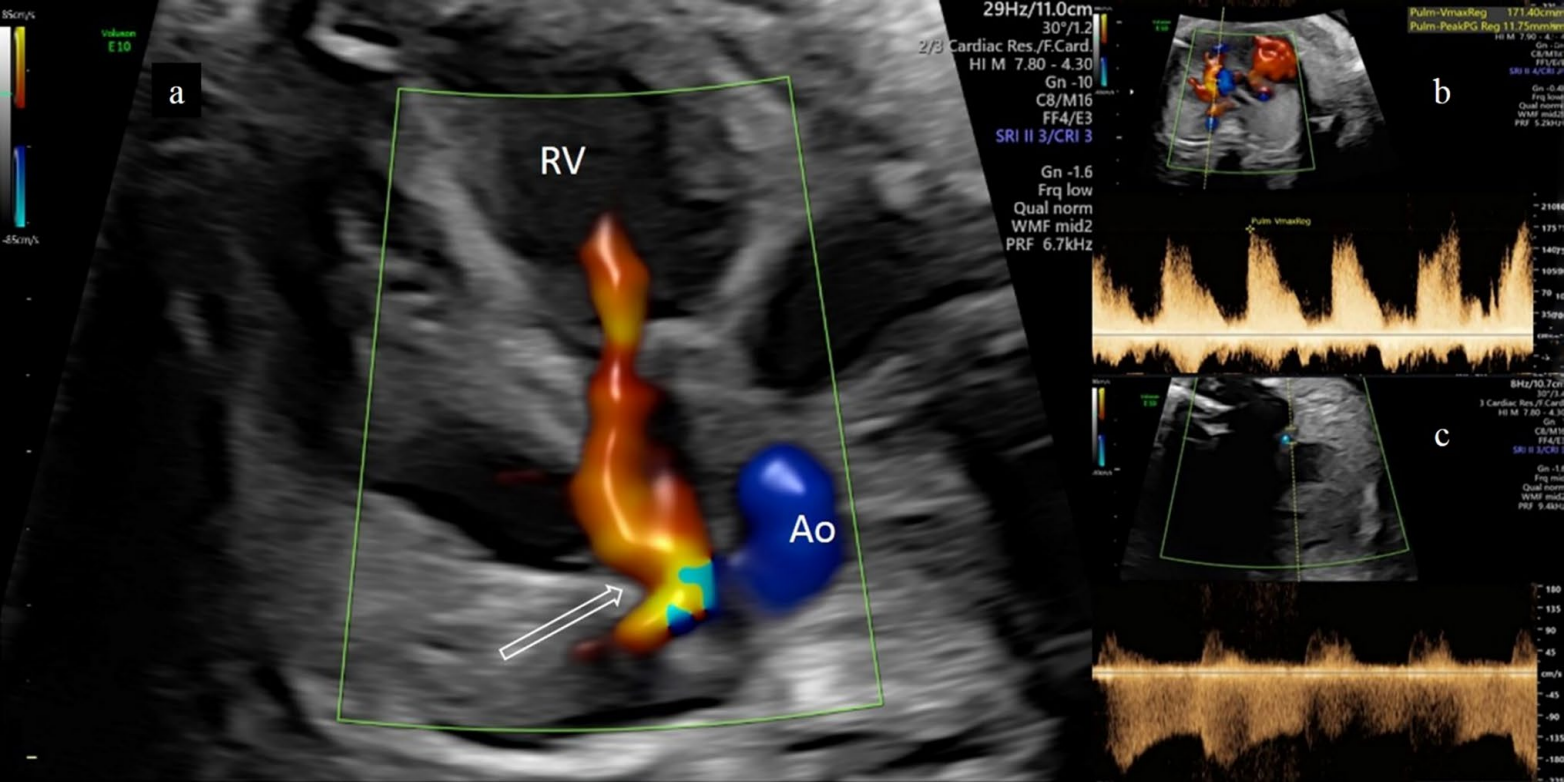
Case 1 **a** Color Doppler during systole at the four chamber view in a fetus with a severe Ebstein anomaly demonstrating a patent ductus arteriosus with a retrograde flow (open arrow) at 31 + 6 weeks gestation. **b** Severe pulmonary regurgitation with a peak velocity of 240 cm/s is shown, leading to a diagnosis of a circular shunt. **c** After 8 days of intrauterine treatment with indomethacin, constriction of a DA is demonstrated on color Doppler with an increase in the diastolic portion of the still reversed blood flow, as well as a peak systolic velocity of > 220 cm/s and distinctly increased diastolic flow with a PI of 0.68

Given the risk of fetal demise, transplacental therapy with indomethacin was discussed to induce DA constriction and started at 31 + 6 weeks of gestation (with 100 mg QID per os [p.o.]). Within 4 days, the DA demonstrated a significant visible narrowing, resulting in turbulent blood flow acceleration with a peak systolic velocity up to 245 cm/s combined with an increase in the diastolic portion (PI 0.68) of the still reversed blood flow (Fig. 3).

Indomethacin was then switched to 400 mg ibuprofen TID p.o. at 33 + 0 weeks and continued until delivery due to the lower reduction in urine output compared to indomethacin with a similar constricting effect on the DA in postnatal studies [15]. Ductal constriction persisted and a resolution of the polyhydramnios and ascites occurred. Blood flow via DA remained retrograde, and there was still no forward flow via the pulmonary valve.

At 37 + 0 weeks, a cesarean section was indicated due to vaginal bleeding. A 2780-g female fetus with Apgar scores of 8, 9, and 9 at 1, 5, and 10 min, respectively, was delivered. Postnatal echocardiography confirmed prenatal findings, showing a massive circular shunt and impaired RV function with an initial oxygen saturation of 80.6%. Initial treatment included ventilatory support with continuous positive airway pressure (CPAP), milrinone (phosphodiesterase-3 inhibitor), and alprostadil (prostaglandin E1) to lower pulmonary artery pressure and keep the patent DA (PDA) open to increase pulmonary blood flow until pulmonary vascular resistance (PVR) naturally decreased. However, a worsening of the TR with a subsequent increased right-to-left shunting at the atrial level occurred, and a lactic acidosis was present on the second day of life. Surgical clipping of the PDA was performed at the 5th day of life. Sustained oxygen desaturation of 50%–60% led to the decision to perform a Starnes procedure, consisting of RV exclusion using a fenestrated path over the tricuspid valve annulus and a placement of a 3.5-mm Blalock–Taussig–Thomas shunt. The patient’s hemodynamics improved, and a biventricular repair using the Da Silva cone technique was performed at 8 months of life. The child is now 14 months old and is in good condition.

Case 2: The second patient was referred to our department at 28 + 3 weeks of gestation because of a suspected cardiac anomaly. Fetal echocardiography showed mild cardiomegaly with a CTAR of 0.55. The RA appeared extremely dilated, and the valve attachment of the tricuspid valve was significantly deeper. Color Doppler imaging revealed a severe holosystolic tricuspid valve regurgitation with a peak velocity of 275 cm/s corresponding to a trans-tricuspid pressure gradient of 30.3 mmHg, and a change in pressure over time (dP/dt) of 474.3 mmHg/s was observed. With the regurgitant jet arising from the center of the right ventricle rather than from the level of the tricuspid valve annulus, a diagnosis of an Ebstein anomaly was made (Celermajer index 1.39). There was antegrade blood flow in the pulmonary trunk with no signs of PR; UA Doppler flow revealed a pulsatility index (UA PI 1.15; 83rd centile) with a positive end-diastolic flow; the patient was therefore scheduled for follow-up 6 weeks later.

**Fig. 4:**
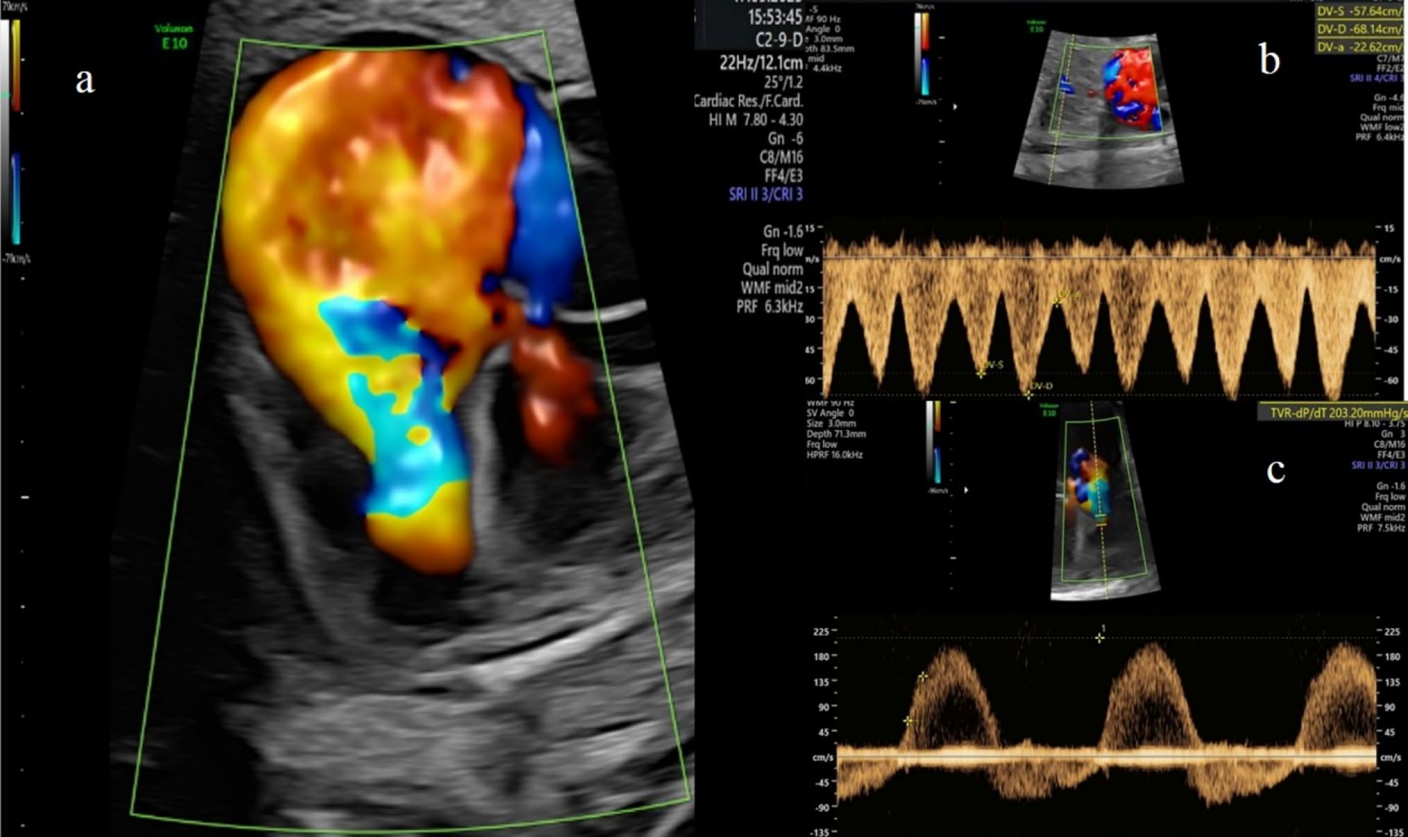
Case 2 **a** Ebstein anomaly at 33 + 0 weeks of gestation, demonstrating massive dilated right atrium with a regurgitant jet arising from the middle of the right ventricle on color Doppler imaging. Color contours appears as “hot air balloon”. **b** Pulsed-wave Doppler of the ductus venosus in the same patient demonstrates a decrease of systolic peak velocity compared to diastolic flow (S < D) and deep v-wave, indicating severe tricuspid insufficiency. **c** Impaired right ventricular function was demonstrated by low peak systolic velocity of 210.8 cm/s and calculating the dP/dt parameter of only 203.2 mmHg/s in tricuspid regurgitation

At 33 + 0 weeks, the fetus had become hydropic, as ascites, skin edema, and polyhydramnios were present. There was progressive cardiomegaly (CTAR 0.69, Celermajer index 1.51), severe PR (peak velocity of 240 cm/s), and a complete retrograde flow via the PDA, displaying a circular shunt. Decrease of systolic flow patterns resulting from the severe tricuspid regurgitation with a low peak systolic velocity of 211 cm/s corresponding to a lowered transtricuspid pressure gradient of only 17.8 mmHg and a change in pressure over time (dP/dt) of only 203.2 mmHg/s caused an abnormal blood flow pattern in the ductus venosus and also in the pulmonary veins with a lower systolic than diastolic peak velocity (S/D ratio 0.84) and deep v-wave (Fig. 4).

Umbilical artery Doppler flow showed an increased pulsatility index (PI 1.15; 94th centile) with a positive end-diastolic flow.

Indomethacin was started 100 mg QID p.o. at 33 + 0 weeks of gestation and switched to ibuprofen (400 mg TID p.o.) after 5 days as DA narrowing was seen. However, hydrops fetalis progressed and indomethacin was restarted 2 days later. No hemodynamically relevant DA constriction was achieved and additionally oligohydramnios occurred. A cesarean section was planned at 35 + 0 weeks due to the severity of the case. A 3630 g-male fetus with Apgar scores of 6, 7, and 8 at 1, 5 and 10 min, respectively, with an umbilical artery pH of 7.31 was delivered. Neonatal echocardiographic findings suggested a high risk of early death, as a massive dilated right atrium bowing into the left atrium, with a diminished flow across the FO, leading to an impaired left ventricular filling, was seen. On the first day of life, an urgent Rashkind procedure was performed and left ventricular function improved. An initial attempt to close the patent PDA with paracetamol failed. Paracetamol was given preference over indomethacin due to impaired renal and cardiac function at that time. Further, right cardiac failure with pulmonary overcirculation occurred and an interventional closure of the PDA by an Amplatzer Vascular Plug II (8 mm × 7 mm) was successfully performed on the 14th day of life. The patient’s hemodynamics improved dramatically by interrupting the circular shunt. Renal function also recovered, and the neonate was discharged on day 59. The child is now 14 months old without a surgical intervention, but tricuspid valve repair by cone procedure is planned.

## Literature research

A systematic review of literature from 2019 to 2023 was performed. PubMed database was reviewed for publications relevant to the following terms: Non-steroidal anti-inflammatory drug therapy or prenatal treatment for severe Ebstein anomaly, prenatal use of indomethacin, DA constriction in fetuses with EA, and circular shunt treatment. Only reports of fetuses with severe Ebstein anomaly causing a circular shunt and being treated with NSAID were included. Duplications were removed. A reference list of selected publications was examined to identify further studies that might be suitable for inclusion. The title and abstract of the retrieved publications were read to assess their relevance. Publications with promising abstracts were full-text assessed for eligibility. The study design and language were not restricted.

Prenatal characteristics and fetal hemodynamic implications of fetuses affected by a severe Ebstein anomaly causing a circular shunt and being treated with NSAID were evaluated for background and prenatal and postnatal data. The following items were considered for assessment: gestational age (GA) at diagnosis, main echocardiographic features as Celermajer index, TR, PR, DA blood flow, ventricular interdependence, UA flow, renal function, amount of amniotic fluid, GA at birth, survival at birth, and postnatal correction.

## Results

Literature review revealed a total of six hits. Only five publications were eligible for data analysis, including three case reports and two case series. Selected studies described a total of 22 pregnancies. Different prenatal echocardiographic findings prior to treatment are summarized in Table 1.

**Table 1:** Prenatal echocardiographic data prior to non-steroidal anti-inflammatory (NSAID) therapy

Author	Study design	Included cases (*n*) treated with NSAID	GA at diagnosis (weeks)	GA at treatment (weeks)	CTR	TR	TR gradient (mmHg)	TR dP/dt (mmHg/s)	Pulmonary valve	Circular shunt type	Ventricular interdependence
Powel (2022) [12]	CR	1	27.3	31.7	NA	Severe	NA	NA	PR	Incomplete	NA
Gill (2021) [11]	CR	1	24.9	33.7	NA	Severe	NA	NA	PR	Complete	NA
Freud (2021) [3]	CS	15	NA	30.3 (22.3–32.2)	0.55 (0.39–0.80)	Severe (100%)	16 (12–25)	NA	PR (100%)	Complete	NA
Lopes (2021) [16]	CR	1	29.0	30	0.5	Severe	NA	NA	PR	Incomplete	+
Torigoe (2019) [17]	CS	4	22.0	30.3	NA	Severe (100%)	27	NA	PR (100%)	Complete	NA
			23.0	25.7	NA		29				
			29.0	32.6	NA		19				
			29.9	34.6	NA		23				
Case 1	CR	1	31.9	31.9	0.84	230 cm/s	22.4	212.4	PR	Complete	+
Case 2	CR	1	28.3	33.0	0.69	275 cm/s	17.9	203.0	PR	Complete	+

*CS* case series, *CR* case report, *CTR* cardio-thoracic ratio, *GA* gestational age, *NA* not available, *PR* pulmonary regurgitation, *TR* tricuspid regurgitation, *TR dP/dt* TR maximum velocity and its change in pressure over time of the TR, + described in the cited literature

Details of prenatal treatment protocols and echocardiographic data during NSAID treatment are shown in Table 2, with postnatal outcomes displayed in Table 3.

**Table 2:** Details of prenatal treatment protocols and echocardiographic data during NSAID treatment (comparing before and after treatment)

Author	Initial indomethacin dosage (mg/day) for DA constriction	Duration of initial treatment (days)	Dosage (mg/day) of maintenance therapy with ibuprofen	Duration of maintenance therapy with ibuprofen (days)	Duration treatment (days)	DA constriction	Abnormal extracardiac Doppler		Hydrops		Oligohydramnios	
							Before NSAID	After NSAID	Before NSAID	After NSAID	Before NSAID	After NSAID
Powel (2022) [12]	100	8	1600	25	33	+	+	−	+	−	−	+
Gill (2021) [11]	100 (reduced on day 3–75 and 50 on day 8 as complete closure of DA was seen)	8	400	21	29	Complete closure	+	−	−	−	NA	NA
Freud (2021) [3]	200–400	3 (1–6)	600–2400	24 (20–105)		12 (80%) constriction (one case had complete closure)	80%	NA	5 (33%)	NA		10 (66.7%)
Lopes (2021) [16]	100 (combined with dipyrone 2 doses/day)	14	300 (combined with Indomethacin 300 mg/day)	14	28	+	NA	NA	+	−	−	+
Torigoe (2019) [17]	100–300	NA	−	−	11	−	+	+	+	+	−	−
	100–300	NA	−	−	49	+	+	−	+	+	−	+
	100–300	NA	1600	10	42	+	+	−	+	−	−	+
	100–300	10	−	−	20	+	−	−	−	−	−	−
Case 1	400	8	1200	28	36	+	+	+	+	−	−	−
Case 2	400	Initial duration for 5 days; restarted as progress occurred with a complete duration of 10 days	1200	5	15	−	+	+	+	+	−	+

*DA* ductus arteriosus, *NA* not available, *NSAID* non-steroidal anti-inflammatory (NSAID) therapy, + described in the cited literature, - absent in the cited literature

**Table 3:** Postnatal outcomes of fetuses after prenatal treatment of severe Ebstein anomaly causing circular shunt

Author	GA at birth (weeks)	Outcome at birth (LB)	DA direct postnatal	Postnatal correction	Renal failure requiring dialysis (*)
Powel (2022) [12]	36.4	+	Left open	None	−
Gill (2021) [11]	38	+	Prenatally complete closure	Starnes procedure	NA
Freud (2021) [3]	35.1 (29.1–39)	12 (80%)	NA	NA	2 (13.3%)
Lopes (2021) [16]	34	+	NA	Initial Starnes procedure (switched to cone repair in the further course)	−
Torigoe (2019) [17]	31.9	LB (but NND in the further course)	Closure tried	NND	−
	32.6	+		Starnes procedure	+ (1–5 days)
	36.0	+		Starnes procedure	+ (1–5 days)
	37.4	+	Closed	None	−
Case 1	37	+	Left open	Starnes procedure	−
Case 2	35	+	Closure	None	−

*DA* ductus arteriosus; *GA* gestational age; *LB* live-born; *NA* not available; *NND* neonatal death; (*): No case of permanent dialysis

## Discussion

While neonatal survival of fetuses affected by Ebstein anomaly significantly depends on the ability to establish adequate pulmonary flow, fetal survival mainly depends on the capability to increase left ventricular volume flow. However, even if the stroke volume of the left ventricle can be sufficiently increased, development of a circular shunt results in a higher risk of low effective combined cardiac output. This leads to a reduced blood flow in the descending aorta, peripheral organs, and the placenta, visible by a distinct decrease in the end-diastolic blood flow in the descending aorta and umbilical artery up to an end-diastolic reverse flow. Increased central venous pressure due to global cardiac failure may result in hydrops fetalis and placentae. Both mechanisms contribute to the high risk of intrauterine fetal death in fetuses with Ebstein anomaly complicated by a circular shunt.

Therefore, detecting a relevant and progressive circular shunt may justify intrauterine treatment in fetuses affected by Ebstein anomaly [4]. We propose an algorithm for prenatal management of patients at risk from the literature and our own experience in Table 4.

**Table 4:** Suggestions for a pragmatic approach to monitor Ebstein anomaly with severe features at risk for circular shunt and NSAID treatment

	Monitoring/therapy	Parameters
Ebstein with severe TR	Monitor every 4 weeks from 20 to 34 weeks	TR velocity, TR (dP/dt), DA flow, presence of PA or PR, DV flow, UA
Detection of functional PA	Monitor every 2 weeks	TR velocity, TR (dP/dt), DA flow, presence of PA or PR, DV flow, UA
Detection of circular shunt with or without hydrops	Initiate indomethacin 100 mg/day, monitor daily or consider increase dose if hydrops is present to 400 mg/day	DA flow (direction and PI), TR velocity, UA, DV flow
(a) Constriction of DA	Switch to ibuprofen 1200–1600 mg/day until delivery, monitor weekly	TR velocity, TR (dP/dt), DA flow, presence of PA or PR, DV flow, UA, amniotic fluid
(b) No change in DA	Increase indomethacin to 400 mg/day	

*DA* ductus arteriosus, *DV* ductus venosus, *PR* pulmonary regurgitation, *TR* tricuspid regurgitation, *TR dP/dt* TR pressure increase over time (mmHg/s), *UA* umbilical artery

Prenatal approaches include expectant management vs. novel attempts with maternal administration of non-steroidal anti-inflammatory drugs (NSAID). In a small case series of prenatal NSAID therapy, higher rates of live birth (86% vs. 67%) and higher rates of survival to hospital discharge (89% vs. 43%) were reported. Therefore, prenatal NSAID therapy should be considered rather than expectant management (or delivery), as also demonstrated in both of our cases [18]. While generally recommended to be initiated at an early stage of systemic steal to prevent brain injury due to hypoperfusion, the presence of a PR with a retrograde ductal flow might be considered as a rationale for initiating intrauterine treatment [19] (Fig. 1).

When functional pulmonary atresia is recorded, i.e., a lack of antegrade flow across the pulmonary valve, which is typically observed prior to the development of PR, serial monitoring at least every 2 weeks should be initiated (Table 4), with focus on the late second trimester as the earliest administration of NSAIDs being given at 22.0 weeks, however, with a median GA at NSAID initiation reported in the literature of 31.8 weeks (range: 30.2–33.2) (Table 2) [3].

In the case of fetal therapy, close monitoring is required and parents must be counseled on the risks of a complete DA closure (9.1%; 2/22), failure of DA constriction (13.6%; 3/22), and the occurrence of oligohydramnios (63.6%; 14/22) as potential side effects of maternal NSAID therapy (Table 2) [3, 18]. However, as renal function impairment occurs mainly temporarily, with no published case of terminal renal insufficiency requiring transplantation or continuous dialysis, renal impairment seems to be negligible [3, 11]. On the contrary, presence of oligohydramnios can also occur in the late stage of fetal cardiac failure, exacerbating the already pre-existing systemic steal phenomenon. With neither duration nor GA at initiating treatment with NSAID correlating with the occurrence of an oligohydramnios, decreased left ventricular function seems to be a more significant parameter attributing to prerenal failure [18].

Regarding the risk of a complete prenatal closure of DA, no complications are suspected during the intrauterine course, while postnatal careful monitoring and a multidisciplinary approach are mandatory, due to neonatal outcome being either improved or worsened.

### Postnatal course

With the onset of breathing, a decreased pulmonary vascular resistance (PVR) and increased pulmonary blood flow require a sufficient RV ejection to allow for an uncomplicated hemodynamic feto-neonatal transition. As in Ebstein anomaly, RV ejection into the pulmonary trunk depends on the interaction of RV function, RV size, and the extent of tricuspid incompetence, and predicting hemodynamic consequences after delivery remains challenging and should take into account: (1) Even if the tricuspid valve is quite incompetent, the volume of regurgitated blood will be small, if the functional RV is small and vice versa; (2) Using color flow mapping, the severity of tricuspid regurgitation can only be estimated semi-quantitatively with limitations. The area and volume of the regurgitated jet are determined by the momentum, probably more by the blood flow velocity than by the volume of the regurgitated blood. In addition, many factors, such as gain, pulse repetition frequency, Doppler frequency, insonation angle, and quality of machine, as well as individual factors, influence the jet expansion in the color flow mapping; (3) Higher oxygen saturation of the pulmonary blood may lower pulmonary arterial vascular resistance and limit smooth muscle development of pulmonary arteries; this may promote a more rapid fall in pulmonary vascular resistance after birth.

Hence, only in fetuses in whom the functional RV cannot develop a pressure high enough to achieve forward flow into the pulmonary trunk, pulmonary perfusion depends exclusively on the ductus arteriosus, and an already closed PDA may lead to cyanosis, necessitating emergency intervention after birth. The two fetuses described in the literature with complete ductal occlusion under NSAID therapy survived [3, 11]. Careful prenatal monitoring and delivery planning prevented adverse outcomes [3, 11]. Further, with both cases being delivered at term, prenatal treatment was able to prolong pregnancy to a more advanced gestational age. Given prematurity being one of the major aspects of perinatal mortality in fetuses affected by EA, even an univentricular palliation must be balanced against being life born (Table 4) [11, 20].

On the contrary, if the PDA is left open, the constant left-to-right shunt prevents the postnatal drop in RV afterload and increases LV volume overload and TR. This prevents antegrade RV output into the pulmonary trunk, further increasing RV afterload and puts the neonate to a higher risk of cardiac failure. In these cases, closure of the PDA is recommended, if RV function is assumed to be sufficient. Nevertheless, as demonstrated in our case, keeping the DA open in the initial course (case 1) deteriorated LV afterload and further aspects of ventricular interdependence.

When evaluating LV function, two mechanisms should be considered: First, the right atrium may become extremely dilated and bow into the left atrium. With the risk of atrial septum abutting the lateral wall of the left atrium, flow cross the foramen ovale (FO) may become diminished with an inadequate right-to-left shunt or FO even becoming restrictive, resulting in a possible impairment of LV volume flow. In these cases, measuring the fossa ovalis/atrial septal length ratio might guide prenatal and postnatal treatment [6, 21]. Second, as a result of septal displacement, an adverse ventriculo-ventricular interaction, known as ventricular interdependence, with paradoxical septal wall motions leading to an abnormal left ventricular geometry, may be present [10]. Causing an additional ineffective volume loading, already impaired LV function might deteriorate and the left myocardial performance index (MPI) might be increased [10].

On the contrary, when evaluating RV function, in tandem with the parameters already mentioned, such as RV ejection, size and extent of tricuspid regurgitation, TR maximum velocity, and its change in pressure over time of the TR (dP/dt) appear to be good indicators of ventricular contractility, predicting RV function and biventricular (BV) outcome. As reported by Ikegawa et al., BV repair seems to be reliably predicted in fetuses with TR maximum velocity > 330 cm/s and is dependent on the change in pressure over time of the TR (dP/dt ≥ 350 mmHg/s) in cases of TR maximum velocity > 240 cm/s and < 330 cm/s [22]. Initial palliated circulation in our first case is in line with the results of Ikegawa et al., although our second case with a dP/dt of 203.2 mmHg/s still resulted in a BV repair [22]. The difference might be explained by the study population by Ikegawa et al. not including fetuses with a circular shunt, or even being treated for them [22]. Reported cutoffs might therefore not be applicable in these cases.

Nevertheless, knowledge of these new approaches is of utmost importance, as previously evaluated scores, such as SAS score or Celermajer index predicting neonatal outcomes, cannot be readily applied to fetuses affected by a circular shunt and routinely measured blood flow parameters of the ductus venosus or the umbilical artery are altered. In consequence, a measured Celermajer index in both of our cases > 1.5 suggested a 100% mortality rate, and a decrease in the venous systolic forward flow of the DV in the second case (S < D) modulated the venous pulsatility index to a more favorable value, although the fetus was still at a high risk for intrauterine demise due to impaired left ventricular function with progressive hydrops fetalis.

In conclusion, our experience demonstrates the complexity of influencing hemodynamic features of prenatal NSAID admission for fetuses affected by Ebstein anomaly presenting with a circular shunt. Evaluation should not only include ductal flow, PR, or regression of a hydrops fetalis but rather ventricular function assessment, as it seems to be an important contributor to outcome. Novel scoring systems such as TRIPP score, including TR maximum velocity, pulmonary artery flow, direction of ductal flow, and left ventricular Tei index, might be helpful [19]. Although changes in ventricular function before and during NSAID treatment for interrupting circular shunt still have to be evaluated in the future, typical Doppler parameters of the DV with a negative a-wave (also quantitative) might be interpreted as an ominous sign for the presence of LV impairment [14, 23]. Changes in the venous ductal blood flow pattern like S < D reversal and systolic notching might be interpreted as severe TR (15), with a potential risk of developing a circular shunt. Prenatal counselling of NSAID treatment of fetuses affected by an Ebstein anomaly must consider neonatal survival being depended on the ability to establish adequate pulmonary flow while fetal survival being strongly linked to compensatory increase left ventricular volume flow. Measurable improvements of hemodynamic parameters during NSAID therapy prenatally, like regress of hydrops fetalis, cannot guarantee a favorable outcome postnatally. As mentioned above, treatment goals differ from the prenatal to postnatal course. Prenatal NSAID treatment appears to improve the initial conditions for postnatal treatment, as reaching a higher gestational age with a higher birth weight also has a significant impact on postnatal treatment. However, postnatal treatment depends on the experience of the pediatric cardiology department and the assessment of the RV function.

In the future, efforts need to be made to agree on which parameters to evaluate in clinical practice to improve the prediction of fetuses at increased risk for circular shunt, consider initiating treatment as soon as circular shunt is detected before hydrops occurs, and find the best treatment regime in terms of drugs and dosage, including long-term follow-up to assess the lasting impact of prenatal treatment on neonatal survival and cardiac function.

## Data Availability

No datasets were generated or analysed during the current study.
